# Amniotic mesenchymal stem cells attenuate diabetic cardiomyopathy by inhibiting pyroptosis via modulation of the TLR4/NF-κb/NLRP3 pathway

**DOI:** 10.3389/fcell.2025.1631973

**Published:** 2025-07-10

**Authors:** Xuan Zhou, Shaoliang Xing, Lina Zhang, Jungu Lu, Deming Li, Yating Wang, Yuhang Ma, Weiqin Chang, Manman Su

**Affiliations:** ^1^ Department of Regenerative Medicine, School of Pharmaceutical Sciences, Jilin University, Changchun, China; ^2^ NMPA Key Laboratory for Quality Control of Cell and Gene Therapy Medicine Products Northeast Normal University, Changchun, China; ^3^ The Second Clinical Hospital, Jilin University, Changchun, China

**Keywords:** diabetic cardiomyopathy, type 2 diabetes mellitus, amniotic mesenchymal stem cells, fibrosis, pyroptosis

## Abstract

Diabetic cardiomyopathy (DCM) is a specific type of cardiac dysfunction in diabetic patients, currently has no effective therapies. The TLR4 signaling pathway, activated through MyD88 and NF-κB, plays a critical role in DCM by triggering the release of pro-inflammatory cytokines and promoting pyroptosis through NLRP3 inflammasomes. Additionally, the TGF-β/Smad signaling pathway drives myocardial fibrosis, further compromising cardiac function. Recently, amniotic mesenchymal stem cells (AMSCs) have emerged as a promising therapeutic option due to their ease of access, low immunogenicity, and ability to differentiate into multiple cell types. In this study, a DCM mouse model was treated with AMSCs via tail vein injection every 2 weeks for four doses. Evaluations included glucose tolerance tests, echocardiography, serum analysis, and histopathological and molecular assessments. Results showed AMSCs improved pancreatic function, reduced blood glucose, and enhanced insulin secretion. Cardiac function and morphology improved, with reduced inflammation. Molecularly, AMSCs inhibited pyroptosis via TLR4/NF-κB/NLRP3 pathway suppression and reduced fibrosis through TGF-β/Smad modulation. These findings indicate AMSCs alleviate DCM cardiac dysfunction and pyroptosis, primarily by inhibiting the TLR4/NF-κB/NLRP3 pathway. The study underscores AMSCs as a promising therapeutic strategy for DCM, warranting further clinical exploration.

## 1 Introduction

Diabetes, a chronic metabolic disorder, has emerged as one of the most significant public health challenges of the 21st century (Zi et al., 2014; Xu et al., 2024). Among diabetic cases, Type 2 Diabetes Mellitus (T2DM) accounts for approximately 90% of all diagnoses. Chronic hyperglycemia associated with diabetes can lead to severe systemic complications, affecting multiple organ systems including the cardiovascular system, eyes, kidneys, nerves, and oral health. Notably, the most detrimental consequence of T2DM is its profound impact on cardiovascular health (He et al., 2025). The relationship between diabetes and cardiac dysfunction was first formally described in 1972 when Rubler et al. reported four diabetic patients who succumbed to chronic heart failure in the absence of traditional risk factors such as hypertension, myocardial ischemia, or other common causes of heart failure (Rubler et al., 1972). This groundbreaking observation laid the foundation for our current understanding of DCM. Today, DCM is clinically defined as cardiac dysfunction occurring in diabetic patients in the absence of other cardiovascular pathologies, including coronary artery disease, uncontrolled hypertension, severe valvular heart disease, and congenital heart defects (Ebrahimi et al., 2025). Despite significant advancements in diabetes management, the pathological mechanisms underlying DCM remain highly complex. Early diagnosis is challenging due to the lack of specific biomarkers, and conventional therapeutic strategies often face limitations due to metabolic contradictions (Tan et al., 2020). Currently has no effective therapies.

The TLR4/NF-κB/NLRP3 pyroptosis axis critically drives DCM pathogenesis. TLR4 hyperactivation initiates MyD88-dependent signaling, promoting MAPK/NF-κB activation and pro-inflammatory mediator upregulation (Peng et al., 2022). Nuclear-translocated NF-κB directly induces NLRP3 transcription (Rezaee et al., 2024),facilitating assembly of the NLRP3 inflammasome (NLRP3/ASC/pro-caspase-1) (Liu et al., 2024). Activated caspase-1 cleaves gasdermin D (GSDMD) to generate pore-forming N-terminal fragments, while concurrently processing pro-IL-1β and pro-IL-18 into mature cytokines (Wang and Hauenstein, 2020; Loveless et al., 2021). This cascade triggers pyroptotic cell death and amplifies inflammation (Lu et al., 2022; Geng et al., 2024). Resultant inflammatory microenvironments establish profibrotic conditions, synergizing with the TGF-β/Smad pathway—a direct mediator of myocardial fibrosis in DCM (Parichatikanond et al., 2020).

Amniotic mesenchymal stem cells (AMSCs), derived from the amniotic membrane of the placenta (Wang et al., 2025), have emerged as a highly promising stem cell population due to their unique advantages, including ease of isolation, low immunogenicity, and robust multi-lineage differentiation potential (Pisheh et al., 2024). These cells have demonstrated significant therapeutic potential across a wide range of medical conditions, including lung injury, cardiac dysfunction, Alzheimer’s disease, kidney injury, stroke, arthritis, and wound healing (Liu et al., 2021; Chen et al., 2015). In the context of diabetes, AMSCs have shown remarkable therapeutic effects in type 2 diabetic mouse models, including the reduction of blood glucose levels, improvement of glucose and lipid metabolism disorders, and attenuation of systemic inflammation (Domínguez-Bendala et al., 2012). A Phase 1 clinical trial report indicates that placenta-derived mesenchymal stem cells (PLMSCs) were used to treat juvenile type 1 diabetes. PLMSCs possess enhanced immunomodulatory and pro-angiogenic capabilities and are suitable for allogeneic transplantation (Madani et al., 2021). Specifically, AMSCs transplantation has been shown to enhance the expression of angiogenic factors in the kidneys, leading to improved glycemic control and amelioration of streptozotocin (STZ)-induced chronic kidney damage (Ni et al., 2023). Furthermore, in diabetic nephropathy, AMSCs have been found to inhibit tubular injury by suppressing the TLR4/NF-κB/NLRP3 signaling pathway (Yuan S. et al., 2022).

Building on these findings, this research seeks to provide a comprehensive understanding of the therapeutic potential of AMSCs in DCM and pave the way for their clinical application in treating this debilitating condition.

## 2 Materials and methods

### 2.1 Cells

The AMSCs were provided by Jilin Zhongke Biotechnology Co., Ltd. The cell product was certified by the National Institutes for Food and Drug Control (Report Numbers: SH202003882, SH202003883, SH202100043, SH202100044, SH202100045, SH202100046). The safety evaluation of intravenous administration was performed by Joinn New Drug Research Center. The sixth-passage cells were used for subsequent experiments.

### 2.2 Mouse DCM model and treatment

**FIGURE 1:**
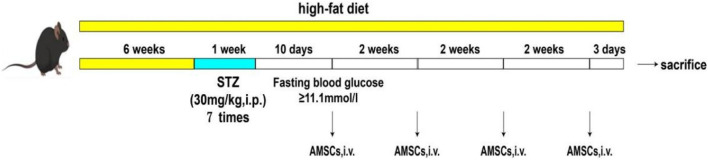
Flowchart of animal model establishment and AMSCs administration. AMSCs, Amniotic mesenchymal stem cells; i.v.,intravenous.

6-week-old male C57BL/6JNifdc mice were purchased from Beijing Vital River Laboratory Animal Technology Co., Ltd. The mice were housed under specific pathogen-free (SPF) conditions with a temperature of 25°C, humidity of 60%, and a 12-h light-dark cycle. The drinking water, feed, bedding, and cages used for housing were all subjected to high-pressure sterilization or irradiation treatment. The mice had *ad libitum* access to water and were provided with abundant food. The high-fat feed containing 60% fat, 20% carbohydrates, and 20% protein was purchased from Jiangsu Collaborative Pharmaceutical and Bioengineering Co., Ltd. The animal experimental procedures were approved by the Animal Management and Ethics Committee of Jilin University (Approval No. 20230089). Following a 3-day acclimatization period, 69 mice were divided into two dietary groups: the experimental group was fed a high-fat diet for 6 weeks, while the normal control group (NC group, n = 10) maintained a standard diet. Starting from the seventh week, mice underwent a 12-h fasting period (with free access to water) and received daily intraperitoneal injections of STZ at 30 mg/kg for seven consecutive days (a cumulative dose of 210 mg/kg over the seven consequitive days). Following each STZ injection, mice received intragastric administration of 20% glucose solution at a dose of 2 g/kg. Ten days after the final STZ injection, fasting blood glucose levels were measured, and mice with blood glucose levels ≥11.1 mmol/L were considered successfully modeled and included in subsequent experiments (Figure 1).

The successfully modeled mice were then randomly allocated into five groups: the model control group (MC group, n = 10), low-dose AMSCs treatment group (L group, n = 10) (1 × 10^6^ cells/kg), medium-dose AMSCs treatment group (M group, n = 10) (2 × 10^6^ cells/kg), high-dose AMSCs treatment group (H group, n = 10) (4 × 10^6^ cells/kg), and metformin control group (Met group, n = 10). The AMSC dosing regimen was established through preliminary safety assessments and pilot studies. AMSCs were administered via tail vein injection every 2 weeks for a total of four treatments. The normal control group and model control group received equivalent volumes of saline via tail vein injection on the same schedule. The metformin control group received daily oral administration of 300 mg/kg metformin for 7 weeks (Ammar et al., 2021; Yan et al., 2021). At the conclusion of the experiment, mice were anesthetized with sodium pentobarbital (60 mg/kg, i.p.) and euthanized by decapitation. Serum samples were collected, and tissue specimens were harvested for subsequent analyses.

### 2.3 CM-Dil-labeled AMSCs tracing

CM-Dil (Thermo Fisher Scientific Inc., United States) stock solution was prepared at a concentration of 1 μg/μL. After digestion, the supernatant of the sixth-passage AMSCs was discarded after centrifugation. Added 1 mL of PBS and 5 μL of CM-Dil stock solution to 5 × 10^6^ cells, mixed well, and incubated at 37°C for 5 min. Then placed it at 4°C for 15 min. CM-Dil-labeled AMSCs were centrifuge at 1000 rpm for 5 min, discarded the supernatant, washed with PBS three times, and resuspended. DCM mice were injected with CM-Dil-labeled AMSCs via tail vein (n = 9, 4 × 10^6^ cells/kg), and the homing of stem cells in the pancreas and heart was examined on the second, third, and seventh days after injection. Mice hearts and pancreases were harvested and embedded in O.C.T., then frozen at −80°C. Frozen tissue blocks were sectioned, stained with DAPI, and observed for the distribution of AMSCs within the tissue.

### 2.4 Blood glucose measurement and oral glucose tolerance test

Fasting blood glucose levels were monitored on a weekly basis using the Accu-Chek glucose meter to track glycemic control throughout the study. Forty-eight hours after the final AMSCs administration, mice were subjected to an oral glucose tolerance test (OGTT) to evaluate glucose metabolism. All mice were fasted for 6 h (8:00 a.m.–2:00 p.m.) (with free access to water), glucose was administered orally at a dose of 2 g/kg. Blood glucose levels were measured at baseline (0 min) and at 30, 60, and 120 min post-glucose administration. The area under the curve (AUC) for blood glucose levels over time was calculated to quantitatively assess glucose tolerance in each group of mice.

### 2.5 Echocardiography

The mice were intraperitoneally injected with 0.3% pentobarbital sodium (0.1 mL/10 g) for anesthesia, and their limbs were fixed. Chest hair was removed using depilatory cream, and coupling agent was applied. Cardiac function in mice from each group was evaluated using the high-frequency probe (40 MHz) of the PanoView β1500 small animal ultrasound imaging system. Measurements of Left Ventricular Internal Dimension Diastole (LVIDd), Left Ventricular Internal Dimension Systole (LVIDs), Left Ventricular Ejection Fraction (LVEF), and Left Ventricular Fraction Shortening (LVFS) were performed across at least three consecutive cardiac cycles.

### 2.6 Inflammatory cytokine and biochemical examination

The commercial assay kits (Nanjing Jiancheng Bioengineering Institute, Nanjing, China) were used to measure serum creatine kinase (CK), creatine kinase-MB (CK-MB), lactate dehydrogenase (LDH), total cholesterol (TC), and triglyceride (TG) in mouse serum. Pancreatic homogenates from mice were prepared, and the insulin (INS) content was determined using an ELISA assay kit (BYabscience, Nanjing, China, BYHS500103). Mice hearts were homogenized, and the levels of TNF-α (Mlbio, Shanghai, China, ml002095), IL-1β (Mlbio, Shanghai, China, ml098416), IL-6 (Mlbio, Shanghai, China, mI098430), IL-10 (Mlbio, Shanghai, China, ml037873), and IL-13 (Mlbio, Shanghai, China, ml106729) were measured using ELISA assay kits.

### 2.7 Histological analysis

The heart and pancreas were fixed, embedded, and sectioned. Both heart and pancreas sections were stained with hematoxylin and eosin (H&E). Masson’s trichrome staining was performed on heart sections to assess the degree of cardiac fibrosis.

### 2.8 Immunohistochemical staining

The heart sections (5 μm) were incubated 15 h at 4°C with primary antibodies against TGF-β (1:100, Beyotime, Shanghai, China, AF0297) and p-smad2/3 (1:100, Beyotime, Shanghai, China, AF5920), respectively, while the pancreatic sections were incubated overnight at 4°C with primary anti-insulin antibody (1:300, Servicebio, CHINA,GB13121-50). Subsequently, all sections were reacted with secondary antibodies at room temperature for 20 min. Following DAB staining and counterstaining, all slides were observed under a microscope.

### 2.9 Immunofluorescence staining

The heart sections were incubated with primary antibodies against TLR4 (1:300, Servicebio, CHINA, GB11519-50) at room temperature for 1 h, followed by incubation with fluorescently labeled secondary antibodies at room temperature in the dark for 1 h. The slides were then covered with a solution containing DAPI and observed under a fluorescence microscope.

### 2.10 Western blot analysis

Total proteins were extracted from heart tissues using RIPA buffer (Beyotime, Shanghai, China, P0013B), and protein concentration was quantified by a BCA protein assay kit (Beyotime, Shanghai, China, P0010). Thirty micrograms of protein was loaded and separated on SDS-PAGE gels and then transferred to a PVDF membrane. Subsequently, the membrane was blocked with 5% non-fat milk and incubated with specific antibody as follows: MyD88 (1:1,000, Cell Signaling Technology, United States), NF-κB p-p65 (1:1,000, Cell Signaling Technology, United States), NF-κB p65 (1:1,000, Cell Signaling Technology, United States), NLRP3 (1:1,000, Cell Signaling Technology, United States), Cleaved-Caspase-1 (1:1,000, Cell Signaling Technology, United States), and GSDMD-N (1:1,000, Affinity Biosciences, United States). Membranes were incubated with HRP-linked secondary antibody, and the Tanon 4600 luminescent image analyzer (Tanon, Shanghai, China) was used for detection. The β-tubulin (Beyotime, Shanghai, China) was used as a loading control.

### 2.11 Statistical analysis

Semi-quantitative analysis of histological results was performed using ImageJ software. The results were expressed as the mean ± standard deviation. Statistical evaluation was performed using GraphPad Prism 10. One-way analysis of variance (ANOVA) with Dunnett’s *post hoc* test was employed for group comparisons. *P* < 0.05 was considered statistically significant.

## 3 Results

### 3.1 AMSCs homing in the hearts and pancreases of DCM mice

**FIGURE 2:**
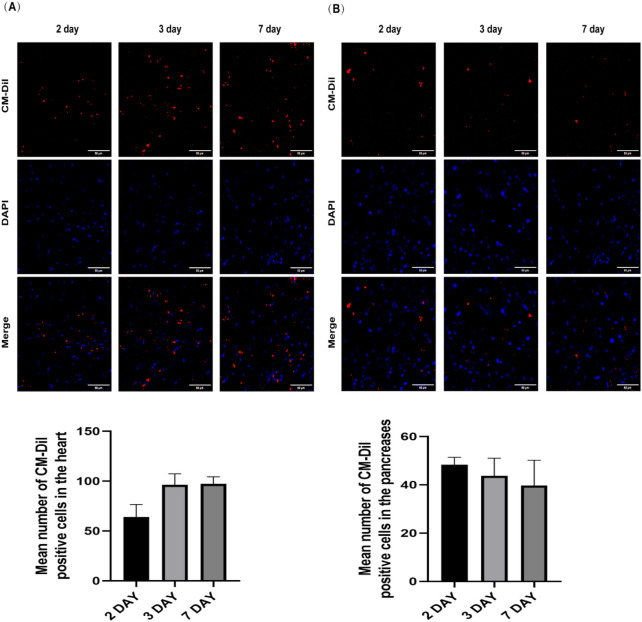
Confocal laser scanning microscopy observation of the distribution of AMSCs in the hearts **(A)** and pancreases **(B)** of DCM mice and quantitative analyses for the count of homed AMSCs (Scale bar = 50 μm).

To track the homing and distribution of AMSCs in DCM mice, CM-Dil-labeled AMSCs were administered via intravenous injection (4 × 10^6^ cells/kg). On days 2, 3, and seven post-injection, frozen sections of the heart and pancreas were prepared and counterstained with DAPI to visualize cell nuclei. As illustrated in Figures 2A,B, AMSCs were observed in both the heart and pancreas of mice from day 2 to day 7 post-injection. Notably, on day 7, a significant number of AMSCs remained localized within the heart tissue, demonstrating their ability to migrate to and persist in target organs. These findings suggest that AMSCs can effectively home to both the heart and pancreas, where they are likely to exert their therapeutic effects. This distribution pattern supports the potential of AMSCs to simultaneously address cardiac dysfunction and pancreatic impairment in DCM.

### 3.2 The effect of AMSCs on glucose and lipid metabolism in DCM mice

**FIGURE 3:**
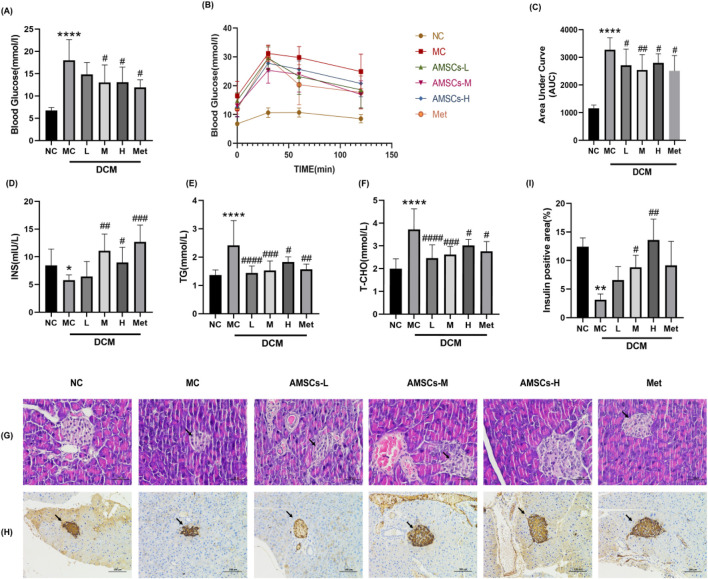
The effect of AMSCs on glucose and lipid metabolism in DCM mice. **(A)** The effect of AMSCs on blood glucose levels in DCM mice. **(B)** Time course of blood glucose changes in mice from each group following glucose loading. **(C)** The effect of AMSCs on the area under the curve (AUC) of blood glucose in DCM mice. **(D)** Insulin levels in pancreatic tissue of DCM mice. **(E)** Serum TG levels in DCM mice. **(F)** Serum TC levels in DCM mice. **(G)** HE staining of pancreatic tissue in DCM mice (Scale bar = 50 μm). **(H)** Immunohistochemical staining of pancreatic insulin in DCM mice (Scale bar = 100 μm) **(I)** Quantitative analysis of insulin expression in the pancreatic. (n = 10, One-way ANOVA with Dunnett’s *post hoc* test, *P < 0.05, **P < 0.01,***P < 0.001, ****P < 0.0001 vs. NC; #*P* < 0.05, ##*P* < 0.01 vs. MC) NC, normal control group; MC,model control group; AMSCs-L, low-dose AMSCs treatment group; AMSCs-M, medium-dose AMSCs treatment group; AMSCs-H, high-dose AMSCs treatment group; Met, Metformin.

Forty-eight hours after the final administration, fasting blood glucose levels were measured in all experimental groups. Compared to the NC group, the MC group exhibited significantly elevated blood glucose levels. Both the AMSCs treatment groups and the Met group showed a marked reduction in blood glucose levels compared to the MC group, indicating that AMSCs effectively lower blood glucose levels in DCM mice (Figure 3A).

To evaluate glucose regulation capacity, an oral glucose tolerance test was performed. As shown in Figure 3B, blood glucose levels in the NC group peaked at 30 min post-glucose administration and subsequently declined, maintaining relatively low levels throughout the test. In contrast, the MC group exhibited a similar peak at 30 min but sustained elevated blood glucose levels over the 120-min period, reflecting impaired glucose tolerance and significant glycemic fluctuations in DCM mice. Both the AMSCs treatment groups and the Met group demonstrated lower blood glucose levels at all time points compared to the MC group. The AUC analysis (Figure 3C) revealed that the AMSCs treatment groups and the Met group had significantly smaller AUCs than the MC group, suggesting that AMSCs substantially improve glucose load capacity in DCM mice. These results collectively demonstrate that AMSCs treatment enhances glucose metabolism in DCM mice.

In T2DM, pancreatic β-cell dysfunction leads to reduced insulin secretion. To assess insulin secretion capacity, pancreatic insulin content was measured. Compared to the NC group, the MC groupexhibited a significant decline in insulin secretion (8.46 ± 2.92 vs. 5.77 ± 0.96, *p* = 0.019),whereas the AMSCs treatment groups and the Met group showed a notable increase in insulin levels relative to the MC group (Figure 3D). These findings indicate that AMSCs therapy can restore pancreatic function in diabetic mice, enhancing pancreatic insulin content and thereby improving glycemic control and glucose tolerance.

Under diabetic conditions, insufficient insulin secretion or insulin resistance impairs glucose utilization, prompting the body to metabolize fats for energy, resulting in elevated serum TC and TG levels. Compared to the NC group, the MC group displayed significantly increased TC (1.994 ± 0.4379 vs. 3.72 ± 0.91, *p* = 0.000) and TG (1.36 ± 0.18 vs. 2.41 ± 0.88, *p* = 0.000) levels, reflecting lipid metabolism dysregulation in DCM mice. However, the AMSCs treatment groups exhibited a significant reduction in serum TC and TG levels compared to the MC group (Figures 3E,F), suggesting that AMSCs therapy can ameliorate lipid metabolism abnormalities in DCM mice.

HE staining and immunohistochemical analysis of mouse pancreatic tissue revealed that in the MC group, both the number and size of islets were reduced (Figure 3G, black arrows), accompanied by slight hyperplasia of surrounding ducts. The insulin-positive staining area was also diminished compared to the NC group. In contrast, AMSCs and Met treatment increased both the number and volume of islets. In the M and H groups, islet morphology was restored, characterized by numerous, regular-shaped islets and normal exocrine tissue resembling serous acinar vesicles. Furthermore, the insulin-positive staining area increased in both the AMSCs and Met groups (Figures 3H,I). These findings demonstrate that AMSCs therapy restores pancreatic function in diabetic mice, increases islet cell mass, and enhances insulin secretory function.

In summary, AMSCs treatment improves glucose metabolism, restores pancreatic function, and corrects lipid metabolism abnormalities in DCM mice, highlighting their therapeutic potential for managing diabetic cardiomyopathy.

### 3.3 The effects of AMSCs on cardiac function and myocardial inflammation in DCM mice

**FIGURE 4:**
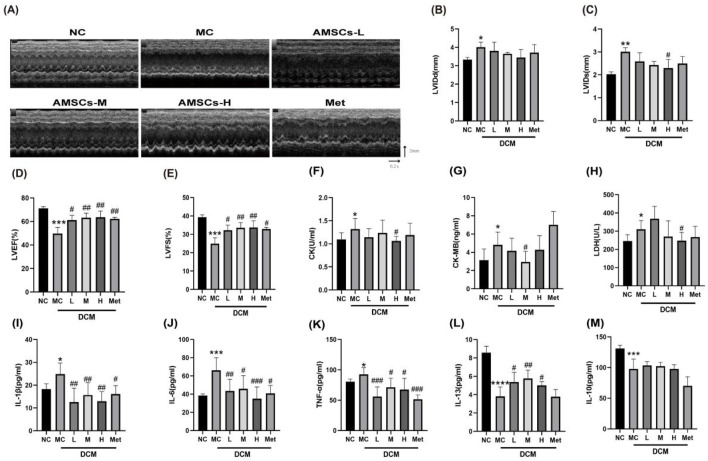
Effects of AMSCs on cardiac function in DCM mice. **(A)** Echocardiography **(B)** LVIDd **(C)** LVIDs **(D)** LVEF **(E)** LVFS **(F)** CK **(G)** CK-MB **(H)** LDH **(I)** IL-1β (n = 9) **(J)** IL-6 (n = 8) **(K)** TNF-α (n = 8) (L) IL-13 (n = 8) (M) IL-10 (n = 7) (One-way ANOVA with Dunnett’s *post hoc* test, *P < 0.05, **P < 0.01, ***P < 0.001, ****P < 0.0001 vs. NC; #*P* < 0.05, ##*P* < 0.01,###*P* < 0.001 vs. MC) NC, normal control group; MC,model control group; AMSCs-L, low-dose AMSCs treatment group; AMSCs-M, medium-dose AMSCs treatment group; AMSCs-H, high-dose AMSCs treatment group; Met, Metformin.

LVEF and LVFS are critical parameters reflecting myocardial contractility and overall cardiac function. A reduction in LVEF and LVFS values typically indicates impaired cardiac function. In DCM, cardiac hypertrophy leads to increased LVIDd and LVIDs. Echocardiographic analysis (Figures 4A–E) revealed that, compared to the NC group, the MC group exhibited significant increases in LVIDd (3.34 ± 0.12 vs. 4.01 ± 0.27, *p* = 0.017) and LVIDs (2.027 ± 0.10 vs. 3.01 ± 0.17, *p* = 0.004), alongside notable decreases in LVEF (71.05 ± 1.61 vs. 49.78 ± 5.17, *p* = 0.000) and LVFS (39.35 ± 1.28 vs. 24.93 ± 3.23, *p* = 0.000), confirming impaired cardiac function in DCM mice. Conversely, the AMSCs treatment groups exhibited a significant decrease in LVIDs and significant increases in LVEF and LVFS. Similarly, the Met group showed significant increases in LVEF and LVFS, suggesting enhanced cardiac function in the mice. Upon myocardial injury, cardiac enzymes are extensively released into the bloodstream. Serum levels of CK, CK-MB, and LDH were measured in mice serum. As shown in Figures 4F–H, compared with the NC group, MC group mice exhibited significantly elevated serum levels of CK (1.10 ± 0.14 vs. 1.32 ± 0.23, *p* = 0.021), CK-MB (3.14 ± 0.95 vs. 4.81 ± 1.41, *p* = 0.046) and LDH (245.70 ± 35.07 vs. 309.80 ± 48.26, *p* = 0.031). Conversely, AMSCs treatment groups showed marked reductions in these cardiac enzymes versus the MC group. Notably, Met group demonstrated no significant differences in enzyme levels compared to the MC group. These findings indicate that AMSCs therapy mitigates myocardial damage in DCM mice.

Inflammation plays a pivotal role in the progression of DCM. Analysis of inflammatory factors in cardiac tissue homogenates revealed that, compared to the NC group, the MC group had significantly elevated levels of pro-inflammatory cytokines, including IL-1β (18.23 ± 2.40 vs. 24.84 ± 4.84, *p* = 0.026), IL-6 (38.52 ± 1.782 vs. 66.23 ± 13.82, *p* = 0.001), and TNF-α (80.40 ± 4.44 vs. 92.61 ± 11.05, *p* = 0.047) (Figures 4I–K). Conversely, the AMSCs treatment groups and the Met group showed a marked reduction in these pro-inflammatory factors. Additionally, the anti-inflammatory cytokine IL-13 was significantly decreased in the MC groupcompared to the NC group (8.56 ± 0.71 vs. 3.81 ± 1.04, *p* < 0.0001), but its expression was restored in the AMSCs and Met treatment groups (Figure 4L). Similarly, the anti-inflammatory cytokine IL-10 was significantly reduced in the MC group, and while the AMSCs treatment groups showed an upward trend in IL-10 expression, the difference was not statistically significant compared to the MC group (Figure 4M).

### 3.4 The effect of AMSCs on Cardiac Histomorphology in DCM mice

**FIGURE 5:**
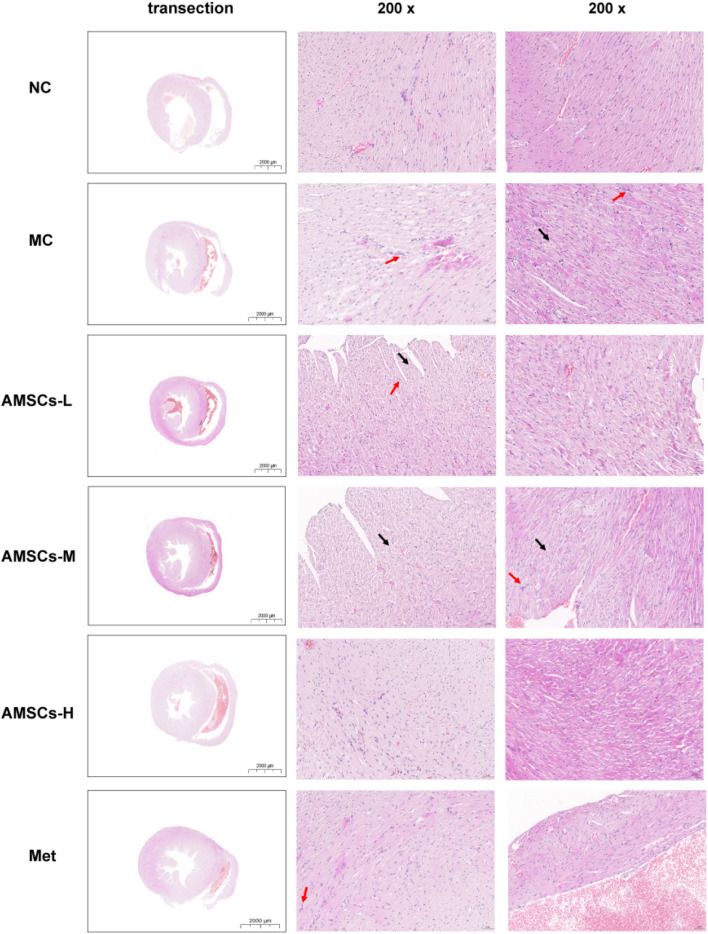
The Effect of AMSCs on Cardiac Histomorphology in DCM Mice (HE staining, low magnification scale bar = 2000 μm, high magnification scale bar = 50 μm) NC, normal control group; MC,model control group; AMSCs-L, low-dose AMSCs treatment group; AMSCs-M, medium-dose AMSCs treatment group; AMSCs-H, high-dose AMSCs treatment group; Met, Metformin.

The HE staining results (Figure 5) provided detailed insights into the structural changes in cardiac tissues across the experimental groups. In the MC group, cardiac tissues exhibited significant pathological alterations, including ventricular dilation, a high incidence of myocardial fiber hydropic degeneration, cellular swelling, and cytoplasmic vacuolation (indicated by black arrows). Mild lymphocyte infiltration (indicated by red arrows) was also observed in the MC group. These findings are consistent with the characteristic myocardial damage observed in DCM. In the L group, myocardial tissues still exhibited ventricular dilation and cytoplasmic vacuolation (indicated by black arrows). However, the extent of myocardial fiber hydropic degeneration and cellular swelling was reduced, primarily localized beneath the endocardium. The M group demonstrated further improvement. We only observed mild myocardial fiber hydropic degeneration and cellular swelling beneath the endocardium, along with cytoplasmic vacuolation (indicated by black arrows). Notably, the H group exhibited near-normal cardiac tissue architecture, characterized by a clear delineation of the endocardium, myocardium, and epicardium. No abnormalities were observed in the myocardial wall or cavity. Myocardial fibers were distinctly separated, consistently arranged, and free of interstitial abnormalities. Similarly, the Met control group exhibited normal myocardial fiber morphology, characterized by clear boundaries and a regular arrangement. These results collectively indicate that AMSC therapy, effectively ameliorates myocardial fiber hydropic degeneration, cellular swelling, cytoplasmic vacuolation, and lymphocyte infiltration in DCM mice. The dose-dependent improvement in cardiac tissue structure underscores the therapeutic potential of AMSCs in mitigating myocardial damage associated with diabetic cardiomyopathy.

### 3.5 The effect of AMSCs on the TGF-β/smad signaling pathway in cardiac tissue of DCM mice

**FIGURE 6:**
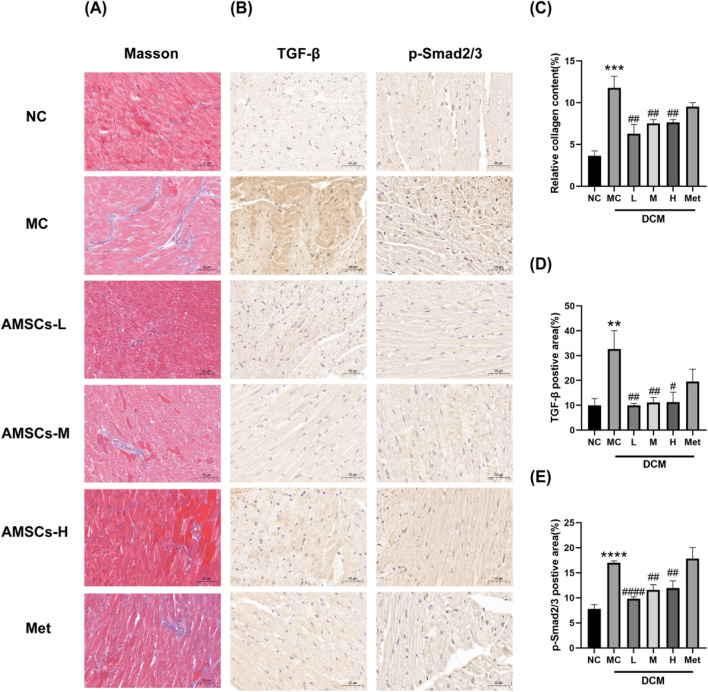
The effect of AMSCs on myocardial fibrosis in DCM mice. **(A)** Masson’s trichrome staining of mouse myocardium (Scale bar = 50 μm). **(B)** Immunohistochemical staining images of TGF-β and p-Smad2/3 (Scale bar = 50 μm). **(C)** Quantitative analysis of Masson’s staining. **(D)** Quantitative analysis of TGF-β expression. **(E)** Quantitative analysis of p-Smad2/3 expression. (n = 3, One-way ANOVA with Dunnett’s *post hoc* test, **P < 0.01, ****P < 0.0001 vs. NC; #*P* < 0.05, ##*P* < 0.01, ####*P* < 0.0001vs MC) NC, normal control group; MC,model control group; AMSCs-L, low-dose AMSCs treatment group; AMSCs-M, medium-dose AMSCs treatment group; AMSCs-H, high-dose AMSCs treatment group; Met, Metformin.

Masson’s trichrome staining, which distinguishes red-stained myocardial fibers from blue-stained collagen fibers, revealed significant differences in cardiac fibrosis among the experimental groups (Figure 6A). Compared to the NC group, the MCl group exhibited exacerbated cardiac fibrosis, characterized by increased collagen deposition. In contrast, the AMSCs treatment groups showed a marked reduction in fibrosis, demonstrating that AMSCs therapy can effectively ameliorate myocardial fibrosis in DCM mice.

During the fibrosis process, the TGF-β/Smad2/3 pathway plays a crucial role. TGF-β activates the Smad signaling pathway intracellularly by binding to its receptor. Upon TGF-β binding to its receptor, receptor activation leads to phosphorylation of Smad2 and Smad3. Phosphorylated Smad2/3 translocates to the nucleus, binding to transcription factors and regulating gene transcription. Activation of the TGF-β/Smad2/3 pathway during fibrosis promotes the transformation and proliferation of fibroblasts. It stimulates the synthesis of collagen and other extracellular matrix proteins, leading to the deposition and accumulation of fibrotic tissue, thus facilitating fibrosis progression (Pan et al., 2023). To investigate the mechanism underlying the anti-fibrotic effects of AMSCs, immunohistochemical staining was performed to assess the expression of TGF-β and phosphorylated Smad2/3 (p-Smad2/3) in cardiac tissues. Compared to the NC group, the MC group exhibited significantly elevated levels of TGF-β and p-Smad2/3, consistent with enhanced fibrotic signaling. However, in the AMSCs treatment groups, the expression of both TGF-β and p-Smad2/3 was significantly reduced compared to the MC group (Figures 6B,C). These findings suggest that AMSCs therapy inhibits the TGF-β/Smad2/3 signaling pathway, thereby suppressing myocardial fibrosis in DCM mice.In summary, AMSCs treatment not only reduces collagen deposition and fibrosis but also modulates key fibrotic signaling pathways, highlighting its potential as a therapeutic strategy to combat myocardial fibrosis in diabetic cardiomyopathy.

### 3.6 The effect of AMSCs on the TLR4//NF-κB/NLRP3 signaling pathway in cardiac tissues of DCM mice

**FIGURE 7:**
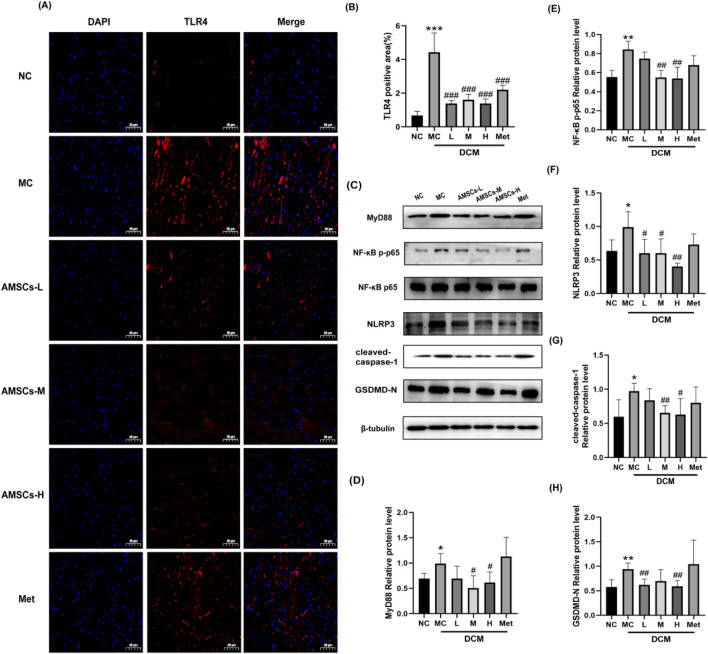
The effect of AMSCs on the expression of essential proteins in the TLR4/NF-κB/NLRP3 signaling pathway in cardiac tissue of DCM mice. **(A,B)** Immunofluorescence and quantification of TLR4. (Scale bar = 50 μm). **(C)** The expression of TLR4/NF-κB/NLRP3 pathway proteins (Western blot analysis). **(D–H)** Quantification of the levels of proteins of B. (n = 3, One-way ANOVA with Dunnett’s *post hoc* test,*P < 0.05, **P < 0.01, ***P < 0.001vs NC; #*P* < 0.05, ##*P* < 0.01, ###*P* < 0.001vs MC) NC, normal control group; MC,model control group; AMSCs-L, low-dose AMSCs treatment group; AMSCs-M, medium-dose AMSCs treatment group; AMSCs-H, high-dose AMSCs treatment group; Met, Metformin.

Cell pyroptosis, as a programmed cell death mode accompanied by an inflammatory response, plays a significant role in the occurrence and development of diabetes and its complications. Excessive activation of the TLR4 signaling pathway links MyD88 to the activation of mitogen-activated protein kinases and NF-κB, leading to the expression of various pro-inflammatory factors. Upon various stimuli, inflammatory cells produce NLRs, such as the NLRP3 inflammasome, which then activates caspase-1, cleaves GSDMD, translocates GSDMD-N to the membrane to form pores, causing cell rupture, ultimately inducing cell pyroptosis (Wang et al., 2019; Ding et al., 2016). To investigate the effects of AMSCs on the TLR4/NF-κB/NLRP3 signaling pathway and pyroptosis in DCM, cardiac tissues from experimental mice were analyzed using immunofluorescence and Western blot techniques. The expression levels of key pathway components, including TLR4, MyD88, NF-κB p-p65, NLRP3, cleaved-caspase-1, and GSDMD-N, were assessed. Immunofluorescence results (Figure 7A) demonstrated that, compared to the NC group, the expression of TLR4 was significantly elevated in the MC group. In contrast, the AMSCs treatment groups exhibited a marked reduction in TLR4 expression relative to the MC group, indicating that AMSCs effectively suppress TLR4 activation. Western blot analysis further corroborated these findings. Compared to the NC group, the MC group showed significantly increased expression levels of MyD88, NF-κB p-p65, NLRP3, cleaved-caspase-1, and GSDMD-N (Figures 7B–G). However, in the AMSCs treatment groups, the expression levels of these proteins were significantly reduced compared to the MC group. Specifically, the downregulation of MyD88, NF-κB p-p65, NLRP3, cleaved-caspase-1, and GSDMD-N suggests that AMSCs inhibit the activation of the TLR4/NF-κB/NLRP3 signaling pathway and suppress pyroptosis. These results collectively indicate that AMSCs treatment attenuates myocardial inflammation and pyroptosis in DCM mice by inhibiting the TLR4/NF-κB/NLRP3 pathway. However, in the Met group, apart from significantly reducing TLR4 expression, no significant inhibitory effect was observed on any other proteins within the TLR4/NF-κB/NLRP3 pathway. This mechanism highlights the therapeutic potential of AMSCs in mitigating cardiac damage associated with diabetic cardiomyopathy, offering a promising strategy for managing this condition.

## 4 Discussion

The complex interplay of metabolic disturbances, chronic inflammation, and oxidative stress in diabetes drives the progression of DCM, making it a significant contributor to cardiovascular morbidity and mortality in diabetic patients. Addressing these underlying mechanisms is essential for developing targeted therapies to prevent or reverse myocardial damage in DCM (Avagimyan et al., 2024). Despite advancements in diabetes management and the development of new antidiabetic medications, severe cardiovascular complications—including ischemic cardiomyopathy and heart failure—continue to significantly impair the quality of life and contribute to high mortality rates among type 2 diabetic patients. Consequently, there is an urgent need to identify and develop therapeutic strategies specifically targeting DCM to address this unmet clinical challenge. Mesenchymal stem cells have emerged as a promising therapeutic option due to their unique properties, including self-renewal, multipotent differentiation capabilities, immunosuppressive effects, and paracrine signaling. Among the various types of MSCs, AMSCs have recently gained attention as a novel and advantageous source. AMSCs can be easily isolated and expanded from the amniotic membrane, a readily available and ethically non-controversial tissue. Their low immunogenicity, high proliferative potential, and robust paracrine activity make them an ideal candidate for regenerative medicine applications. AMSCs hold particular promise for treating DCM due to their ability to modulate inflammation, reduce fibrosis, and promote tissue repair. By targeting key pathological mechanisms such as oxidative stress, inflammation, and myocardial remodeling, AMSCs offer a multifaceted approach to improving cardiac function in diabetic patients. As research continues to uncover their therapeutic potential, AMSCs may represent a breakthrough in the treatment of diabetic cardiomyopathy and its associated complications (Ghamari et al., 2020).

The TLR4/NF-κB/NLRP3 signaling pathway serves as a core signaling network linking the innate immune system to inflammatory responses, with its activation and regulation involving multi-layered molecular interactions. A meta-analysis indicates that the TLR4 signaling pathway plays a pivotal role in the pathogenesis of DCM (Yuan J. X. et al., 2022). Under hyperglycemic conditions, the TLR4/NF-κB/NLRP3 signaling pathway is aberrantly activated, driving the onset and progression of DCM through multiple mechanisms.

Pyroptosis, a newly identified form of programmed cell death, plays a critical role in DCM pathogenesis. In the hyperglycemic milieu, activated NLRP3 inflammasome cleaves caspase-1. Activated caspase-1 not only catalyzes the maturation of pro-IL-1β and pro-IL-18 but also specifically cleaves GSDMD, generating its GSDMD-N. The GSDMD-N domain forms pores in the cell membrane, leading to osmotic lysis and cell death. Within myocardial tissue, pyroptotic cells release large quantities of damage-associated molecular patterns (DAMPs), such as HMGB1 (High Mobility Group Box 1) and ATP (Adenosine Triphosphate), which further amplify the inflammatory response, establishing a vicious cycle of “inflammation-cell death-more inflammation” (Jiang et al., 2025; Li et al., 2025). Our study demonstrates that AMSCs suppress the activation of the TLR4/MyD88 pathway, leading to downregulation of NF-κB activity and subsequent upregulation of the NLRP3 inflammasome. This consequently inhibits the cleavage of caspase-1, reduces the generation of the GSDMD-N terminal fragment, and ultimately prevents the occurrence of cardiomyocyte pyroptosis.

A study have shown that NLRP3 inflammasome activation leads to the release of IL-1β and IL-18 (WANG, 2024). Among these, IL-1β stimulates fibroblasts to secrete TGF-β1, which subsequently phosphorylates Smad2/3. Our results demonstrate that diabetic mice exhibit elevated myocardial expression of IL-1β. Following AMSC treatment, however, the expression of both TGF-β and p-Smad2/3 was reduced in the myocardium. This indicates that AMSCs can suppress the inflammation-induced expression of TGF-β/Smad2/3.

This study has several limitations. First, our conclusions are drawn from a small sample size; therefore, larger-scale studies are necessary for broader validation before clinical application. Second, additional *in vitro* experiments should be conducted on AMSCs, such as investigating the effects of high-glucose culture medium on the immune behavior and secretory functions of cultured AMSCs, to further elucidate their cellular biology under such conditions. Finally, to gain deeper insights into how AMSCs exert optimal therapeutic effects in DCM, more comprehensive long-term studies focusing on delivery routes, potential immunogenicity, and long-term safety are warranted.

## 5 Conclusion

In summary, AMSCs demonstrate significant therapeutic potential in DCM by addressing multiple pathological mechanisms. AMSCs improve insulin secretion capacity and ameliorate glucose and lipid metabolism disorders through the repair of pancreatic injury in DCM mice. Furthermore, AMSCs attenuate cardiac dysfunction and inhibit pyroptosis in the myocardium, with the suppression of the TLR4/NF-κB/NLRP3 pathway playing a pivotal role in these protective effects. By targeting inflammation, fibrosis, and cell death pathways, AMSCs offer a comprehensive approach to mitigating the progression of DCM. These findings position AMSCs as a promising future therapeutic strategy for the treatment of diabetic cardiomyopathy, potentially improving outcomes for patients with this debilitating condition.

## Data Availability

The original contributions presented in the study are included in the article/supplementary material, further inquiries can be directed to the corresponding authors.
